# Preoperative Lymphocyte-to-Monocyte Ratio as a Prognostic Predictor of Long-Term Mortality in Cardiac Surgery Patients: A Propensity Score Matching Analysis

**DOI:** 10.3389/fcvm.2021.639890

**Published:** 2021-02-22

**Authors:** Zhuoming Zhou, Mengya Liang, Huawei Wu, Suiqing Huang, Rennan Weng, Jian Hou, Zhongkai Wu

**Affiliations:** ^1^Department of Cardiac Surgery, First Affiliated Hospital of Sun Yat-sen University, Guangzhou, China; ^2^NHC Key Laboratory of Assisted Circulation, Sun Yat-sen University, Guangzhou, China; ^3^Department of Neurobiology, Physiology and Behavior, College of Biological Sciences, University of California, Davis, Davis, CA, United States

**Keywords:** cardiopulmonary bypass, cardiac surgery, lymphocyte, monocyte, propensity score matching

## Abstract

**Aims:** To evaluate the prognostic value of the preoperative lymphocyte-to-monocyte ratio (LMR) in patients who underwent cardiac surgery.

**Methods:** Clinical data were extracted from the Medical Information Mart for Intensive Care III (MIMIC-III) database. The optimal cutoff value of LMR was determined by X-tile software. The Cox proportional hazard model was applied for the identification of independent prognostic factors of 4-year mortality and survival curves were estimated using the Kaplan-Meier method. In order to balance the influence of potential confounding factors, a 1:1 propensity score matching (PSM) method was performed.

**Results:** A total of 1,701 patients were included. The X-tile software indicated that the optimal cutoff value of the LMR for 4-year mortality was 3.58. After PSM, 489 pairs of score-matched patients were generated. The Cox proportional hazard model showed that patients with an LMR < 3.58 had a significantly higher 4-year mortality than patients with an LMR ≥ 3.58 in the entire cohort (HR = 1.925, 95%CI: 1.509–2.456, *p* < 0.001) and the PSM subset (HR = 1.568, 95%CI: 1.2–2.05, *p* = 0.001). The survival curves showed that patients with an LMR < 3.58 had a significant lower 4-year survival rate in the entire cohort (71.7 vs. 88.5%, *p* < 0.001) and the PSM subset (73.2 vs. 81.4%, *p* = 0.002).

**Conclusions:** A lower LMR (<3.58) was associated with a higher risk of 4-year mortality and can serve as a prognostic predictor of the long-term mortality in cardiac surgery patients.

## Introduction

Cardiac surgery, especially with cardiopulmonary bypass (CPB), can provoke a nonspecific inflammatory response, and inflammatory cells play a vital role in the development of inflammation both preoperatively and postoperatively (1). Inflammatory cells, such as neutrophils, lymphocytes and monocytes, are considered typical inflammatory biomarkers and has been verified to have a close relationship with cardiovascular diseases like atherosclerosis and heart failure. These cells not only can induce diseases but can also predict cardiovascular prognosis (2, 3).

Recently, some markers composed of the ratios of white blood cell (WBC) subgroups have been newly introduced and demonstrated to be associated with the risks and outcomes of cardiovascular diseases and related surgeries. For example, for cardiac surgery patients, the value of the neutrophil-to-lymphocyte ratio (NLR) can serve as a predictor of early and late mortality (4, 5), postoperative acute kidney injury (6), and postoperative atrial fibrillation (7, 8), while the platelet-to-lymphocyte ratio (PLR) has been reported to be a predictor of prognosis in patients with aortic dissection (9).

The lymphocyte-to-monocyte ratio (LMR), a novel emerging inflammatory marker, has been shown to be independently related to the outcomes of various cardiovascular diseases, including coronary artery disease (10) and heart failure (11). The LMR has also been demonstrated to be associated with the in-hospital mortality of patients with acute type A aortic dissection (12) and saphenous vein graft disease in patients who underwent coronary artery bypass grafting (CABG) (13). However, currently no researches reported the association between LMR and the long-term survival for patients undergoing cardiac surgery.

In the present study, we aim to investigate the association between the LMR and the risk of mortality in patients who underwent on-pumped cardiac surgery during a 4-year follow-up.

## Methods

### Data Source

Data were obtained from the Medical Information Mart for Intensive Care III (MIMIC-III) database, which is a large, freely available database with more than 40,000 patients who had critical care unit stays at the Beth Israel Deaconess Medical Center between 2001 and 2012, of which patients in the CareVue system were followed for at least 4-year (14). Our right to access the database and acquire the data were approved by the institutional review boards of the Massachusetts Institute of Technology (Cambridge, MA, USA) after one of our authors (Zhou) finished the online training for the Collaborative Institutional Training Initiative (CITI) program of the National Institutes of Health (NIH) (Record ID 35971811).

### Patient Selection

Of all patients in the MIMIC-3 database, we included patients as follows: (1) those who underwent on-pump cardiac surgery; (2) age older than 18 years; (3) have full records of routine preoperative blood examinations within the first 24 h of admission. Normal values of monocyte counts were defined as between 0.12^*^10^9^/L and 0.8^*^10^9^/L and lymphocyte counts were defined as between 0.8^*^10^9^/L and 4.0^*^10^9^/L.

### Data Extraction

All data was inquired and extracted using the Structured Query Language (SQL), and pgAdmin4 for PostgreSQL was used as the administrative platform. The extracted data included: (1) demographics: age, gender, and ethnicity; (2) vital signs: heart rate (HR), systolic blood pressure (SBP), diastolic blood pressure (DBP), respiratory rate (RR), temperature and percutaneous oxygen saturation (SpO_2_); (3) comorbidities: congestive heart failure (CHF), cardiac arrhythmias, valvular disease, hypertension, chronic pulmonary disease, renal failure, liver disease, coagulopathy and diabetes; (4) laboratory events: peripheral white blood cell count, neutrophil count, monocyte count, lymphocyte count, platelet count, hemoglobin, serum sodium, serum potassium, serum creatinine and LMR (calculated by dividing the lymphocyte count by the monocyte count; (5) SAPS II and SOFA scores; (6) surgical type: coronary bypass artery grafting (CABG). ICU length of stay, 90-day mortality and 4-year mortality were recorded in order to analyze the outcomes. Considering the proportion of missing data for each variable was <1.5%, we omitted them directly in further analysis.

### Propensity Score Matching

Considering the patient selection criteria can hardly be completely random, we applied the propensity score matching (PSM) method to balance the influence of selection bias and potential confounding factors. The PSM analysis was based on the logistic regression model, and the propensity score was calculated according to the following baseline characteristics: age, gender, ethnicity, CHF, cardiac arrhythmias, valvular disease, hypertension, chronic pulmonary disease, renal failure, liver disease, peripheral white blood cell count, platelet count, hemoglobin, serum sodium, serum potassium, serum creatinine and SAPS II score. Pairs of patients with LMR< 3.58 or ≥ 3.58 were derived using 1:1 matching with a caliper of 0.02. Eventually, a total of 978 patients were propensity score-matched and 489 pairs of score-matched patients were generated.

### Statistical Analysis

Continuous variables were presented as the mean ± SD or median (interquartile range) and compared by *t*-test or Mann-Whitney U test. Categorical data were presented as numbers with proportions and analyzed by χ2 test. After PSM, the paired *t*-test and Wilcoxon rank sum test for continuous data and the McNemar test for categorical data was used for comparison between the matched groups. The optimal cutoff value of the LMR for 4-year mortality was determined by X-tile (Version 3.6.1, Yale University School of medicine) software (15). Survival curves were estimated using the Kaplan-Meier method and compared by the log-rank test. Receiver operating characteristic (ROC) curves were constructed, and the area under the curve (AUC), sensitivity and specificity were calculated.

The Cox proportional hazard model was applied for the univariate and multivariate analyses to identify independent prognostic factors of 4-year mortality after cardiac surgery. To evaluate the association between the LMR and mortality, model 1 was adjusted for age, gender, CHF, cardiac arrhythmias, valvular disease, hypertension, renal failure and liver disease; model 2 was adjusted for age and gender. The results are presented as hazard ratios (HRs) and 95% confidence intervals (CIs). Subgroup analyses were performed with Cox regression model according to age strata (<70 and ≥70 years), gender, liver disease, renal failure, hypertension, diabetes, chronic pulmonary disease, CHF, cardiac arrhythmias, valvular disease and CABG. All tests were two-sided, and *p*-values <0.05 were considered significant. All statistical analyses in our study were performed using STATA V.14.0, SPSS Statistics 22 (IBM, Chicago, IL) and GraphPad Prism 8.

## Results

### Characteristics of Patients

In total, 1,701 patients who met the selection criteria were enrolled in our study. Based on the results calculated by the X-tile software, the optimal cutoff value of preoperative LMR for 4-year mortality was set as 3.58 (with a sensitivity of 71.1% and a specificity of 56.6%) and divided all patients into two groups according to the LMR: < 3.58 (*n* = 571) and ≥ 3.58 (*n* = 1,130). The baseline characteristics of enrolled patients are briefly summarized in the Table 1, including demographics, vital signs, laboratory events, comorbidities and scores.

**Table 1:** Baseline characteristics before propensity score matching (*n* = 1,701).

**Characteristics**	**LMR**		
	**<3.58 (*n* = 571)**	**≥3.58 (*n* = 1,130)**	***p*-value**
**Demographics**			
Age, years	69.3 (61.2–77)	65.8 (56.3–74.3)	<0.001
Male, *n* (%)	422 (73.9)	756 (66.9)	0.003
Ethnicity, *n* (%)			0.008
White	428 (75)	816 (72.2)	
Black	10 (1.8)	54 (4.8)	
Others	133 (23.3)	260 (23)	
**Vital signs**			
HR, beats/minute	85.1 (79.5–91.4)	85.4 (79.7–91.4)	0.640
SBP, mmHg	111.7 (106.1–120)	111.3 (105.7–119.1)	0.299
DBP, mmHg	55.8 (51.7–60.2)	56.6 (52.7–61)	0.016
RR, times/minute	17 (15.5–19.4)	16.7 (15.1–18.8)	0.003
Temperature, °C	36.9 (36.6–37.2)	36.9 (36.6–37.2)	0.321
SpO2, %	98.2 (97.4–99)	98.4 (97.5–99.1)	0.048
**Comorbidities**			
Congestive heart failure	267 (46.8)	297 (26.3)	<0.001
Cardiac arrhythmias	343 (60.1)	493 (43.6)	<0.001
Valvular disease	169 (29.6)	225 (19.9)	<0.001
Hypertension	380 (66.5)	793 (70.2)	0.127
Chronic pulmonary disease	117 (20.5)	164 (14.5)	0.002
Renal failure	96 (16.8)	70 (6.2)	<0.001
Liver disease	24 (4.2)	30 (2.7)	0.850
Coagulopathy	45 (7.9)	58 (5.1)	0.025
Diabetes	188 (32.9)	397 (35.1)	0.365
**Laboratory events**			
LMR	2.6 (2–3.1)	5.4 (4.5–7)	<0.001
NLR	5.6 (3.8–9.2)	2.5 (1.8–3.6)	<0.001
PLR	181.7 (137.5–255.1)	120.5 (92.4–156.7)	<0.001
WBC, 10^9^/L	8.9 (6.7–12.2)	7.5 (6–9.4)	<0.001
Neutrophils, %	77.2 (70.1–84.1)	66 (59.1–72.8)	<0.001
Monocytes, %	5.5 (4.2–7)	4.6 (3.7–5.5)	<0.001
Lymphocytes, %	13.7 (9–18.7)	25.7 (20.4–31.8)	<0.001
Platelets, 10^9^/L	221 (175–282)	228 (190.8–275.3)	0.056
Hemoglobin, g/Dl	10.1 (9.2–11.1)	10.5 (9.5–11.5)	<0.001
Serum sodium, mmol/L	137.5 (135.5–139)	137.5 (136–139)	0.013
Serum potassium, mmol/L	4.6 (4.2–4.9)	4.5 (4.2–4.8)	0.033
Serum creatinine, mg/Dl	1 (0.8–1.4)	0.9 (0.7–1.1)	<0.001
**Scores**			
SAPS II	36 (29–43)	31 (25–39)	<0.001
SOFA	5 (3–7)	4 (3–6)	<0.001
**Surgical type**			
CABG	390 (68.3)	837 (74.1)	0.012

*Values are presented as the mean ± standard deviation, median (interquartile range), or number of patients (%)*.

*HR, heart rate; SBP, systolic blood pressure; DBP, diastolic blood pressure; RR, respiratory rate; SpO2, percutaneous oxygen saturation; LMR, lymphocyte-to-monocyte ratio; NLR, neutrophil-to-lymphocyte ratio; PLR, platelet-to-lymphocyte ratio; WBC, white blood cell; SAPS II, Simplified Acute Physiology Score II; SOFA, Sequential Organ Failure Assessment; CABG, coronary artery bypass grafting*.

Significant differences in baseline characteristics could be observed between the two groups. Patients with a lower LMR tended to be older and male with a lower DBP, SpO2, lymphocyte count, hemoglobin, and serum sodium and higher NLR, PLR, WBC count, neutrophil count, monocyte count, serum potassium, serum creatinine, SAPS II score, and SOFA score, as well as a history of CHF, cardiac arrhythmias, valvular disease, chronic pulmonary disease, renal failure, liver disease, and coagulopathy, while tending not to have hypertension and diabetes.

### The Prognostic Significance of LMR Before PSM

Compared with patients with LMR ≥ 3.58, patients with LMR < 3.58 were at higher risk of prolonged ICU stay (3.1 vs. 2.2 days, *p* < 0.001), hospital mortality (4.73 vs. 1.77%, *p* < 0.001), 30-day mortality (6.48 vs. 1.86%, *p* < 0.001),90-day mortality (10.3 vs. 2.9%, *p* < 0.001) and 4-year mortality (28.9 vs. 11.5%, *p* < 0.001) (Table 2).

**Table 2:** Outcomes of patients before and after PSM matched and patients with normal lymphocyte and monocyte counts.

	**LMR < 3.58**	**LMR ≥ 3.58**	***p*-value**
**Before PSM**			
	*N* = 571	*N* = 1,130	
ICU stay, days	3.1 (1.9-5.6)	2.2 (1.3–4)	<0.001
Hospital mortality, *n* (%)	27 (4.73)	20 (1.77)	<0.001
30-day mortality, *n* (%)	37 (6.48)	21 (1.86)	<0.001
90-day mortality, *n* (%)	59 (10.3)	33 (2.9)	<0.001
4-year mortality, *n* (%)	165 (28.9)	130 (11.5)	<0.001
**After PSM**			
	*N* = 489	*N* = 489	
ICU stay, days	3.0 (1.7–5.2)	2.9 (1.7–5.3)	0.003
Hospital mortality, *n* (%)	17 (3.48)	13 (2.66)	0.458
30-day mortality, *n* (%)	24 (4.91)	15 (3.07)	0.141
90-day mortality, *n* (%)	42 (8.6)	24 (4.9)	0.027
4-year mortality, *n* (%)	131 (26.8)	91 (18.6)	0.002
**Normal lymphocytes and monocytes group**			
	*N* = 393	*N* = 1,058	
ICU stay, days	3.0 (1.6–5.2)	2.2 (1.3–3.9)	<0.001
Hospital mortality, *n* (%)	19 (4.77)	16 (1.52)	<0.001
30-day mortality, *n* (%)	26 (6.53)	18 (1.71)	<0.001
90-day mortality, *n* (%)	37 (9.3)	29 (2.8)	<0.001
4-year mortality, *n* (%)	111 (27.9)	112 (10.6)	<0.001

*Values are presented as the median (interquartile range), or number of patients (%)*.

*LMR, lymphocyte-to-monocyte ratio; PSM, propensity score matching*.

A univariate Cox regression analysis was conducted to select the variables of prognostic value for 4-year mortality, and age (*p* < 0.001), gender (*p* < 0.001), CHF (*p* < 0.001), cardiac arrhythmias (*p* < 0.001), valvular disease (*p* < 0.001), hypertension (*p* = 0.008), renal failure (*p* < 0.001), and liver disease (*p* < 0.001) were selected to be adjusted in the multivariate Cox regression analysis. The results of the univariate and multivariate analyses are summarized in Table 3. In the multivariate analysis, Model 1 was adjusted for age, gender, CHF, cardiac arrhythmias, valvular disease, hypertension, renal failure and liver disease, while Model 2 was adjusted for age and gender. Patients with an LMR < 3.58 had significantly higher 4-year mortality compared to patients with an LMR ≥ 3.58 (Model 1: HR = 1.925, 95%CI: 1.509–2.456, *p* < 0.001; Model 2: HR = 2.651, 95%CI: 2.075–3.31, *p* < 0.001).

**Table 3:** Univariate and multivariate Cox regression analyses for 4-year mortality in cardiac surgery patients.

	**Univariate analysis**		**Multivariate analysis**			
			**Model 1**		**Model 2**	
	***p***	**HR (95%CI)**	***p***	**HR (95%CI)**	***p***	**HR (95%CI)**
	**Unmatched group (*****n*****= 1,701)**					
LMR < 3.58	<0.001	2.818 (2.239–3.546)	<0.001	1.925 (1.509–2.456)	<0.001	2.621 (2.075–3.310)
Age	<0.001	1.05 (1.038–1.061)	<0.001	1.042 (1.030–1.054)	<0.001	1.041 (1.030–1.052)
Gender (male)	<0.001	0.628 (0.497–0.793)	0.004	0.700 (0.550–0.891)	0.001	0.670 (0.527–0.851)
CHF	<0.001	2.278 (1.813–2.862)	0.008	1.380 (1.086–1.754)		
Arrhythmia	<0.001	2.074 (1.632–2.637)	0.020	1.348 (1.048–1.735)		
Valvular disease	<0.001	2.188 (1.729–2.770)	<0.001	1.656 (1.303–2.104)		
Hypertension	0.008	0.726 (0.574–0.920)	<0.001	0.592 (0.461–0.759)		
Renal failure	<0.001	3.103 (2.355–4.087)	<0.001	2.412 (1.791–3.248)		
Liver disease	<0.001	3.727 (2.473–5.616)	<0.001	3.580 (2.350–5.454)		
	**Matched group (*****n*** **= 978)**					
LMR < 3.58	0.002	1.533 (1.173–2.003)	0.002	1.517 (1.159-1.986)	0.001	1.568 (1.200–2.050)
Age	<0.001	1.043 (1.029–1.057)	<0.001	1.042 (1.028–1.056)	<0.001	1.038 (1.024–1.052)
Gender (male)	<0.001	0.476 (0.363–0.623)	0.004	0.656 (0.494–0.872)	<0.001	0.581 (0.440–0.767)
CHF	0.001	1.580 (1.215–2.056)	0.041	1.323 (1.012–1.729)		
Arrhythmia	0.001	1.625 (1.225–2.157)	0.099	1.280(0.955–1.715)		
Valvular disease	<0.001	1.690 (1.283–2.227)	0.005	1.493 (1.130–1.973)		
Hypertension	0.040	0.754 (0.575–0.988)	0.003	0.649 (0.489–0.862)		
Renal failure	<0.001	2.190 (1.582–3.030)	<0.001	2.250 (1.603–3.158)		
Liver disease	<0.001	2.967 (1.783–4.935)	<0.001	3.444 (2.038–5.818)		
	**Normal lymphocytes and monocytes group (*****n*** **= 1,451)**					
LMR < 3.58	<0.001	2.923 (2.248–3.801)	<0.001	2.052 (1.553–2.712)	<0.001	2.656 (2.032–3.471)
Age	<0.001	1.050 (1.037–1.064)	<0.001	1.043 (1.029–1.057)	<0.001	1.041 (1.028–1.055)
Gender (male)	0.002	0.659 (0.503–0.863)	0.045	0.748 (0.563–0.993)	0.021	0.718 (0.543–0.951)
CHF	<0.001	2.551 (1.962–3.317)	0.003	1.515 (1.147–2.003)		
Arrhythmia	<0.001	2.056 (1.564–2.704)	0.087	1.286 (0.964–1.715)		
Valvular disease	<0.001	2.065 (1.567–2.722)	0.005	1.500 (1.130–1.991)		
Hypertension	0.019	0.720 (0.548–0.947)	<0.001	0.510 (0.380–0.684)		
Renal failure	<0.001	3.479 (2.539–4.767)	<0.001	2.562 (1.814–3.618)		
Liver disease	<0.001	3.447 (2.072–5.733)	<0.001	3.555 (2.092–6.042)		

*Model l was adjusted for age, gender, CHF, cardiac arrhythmias, valvular disease, hypertension, renal failure and liver disease*.

*Model 2 was adjusted for age and gender*.

*HR, hazard ratio; CI, confidence interval; LMR, lymphocyte-to-monocyte ratio; CHF, congestive heart failure*.

**Figure 1:**
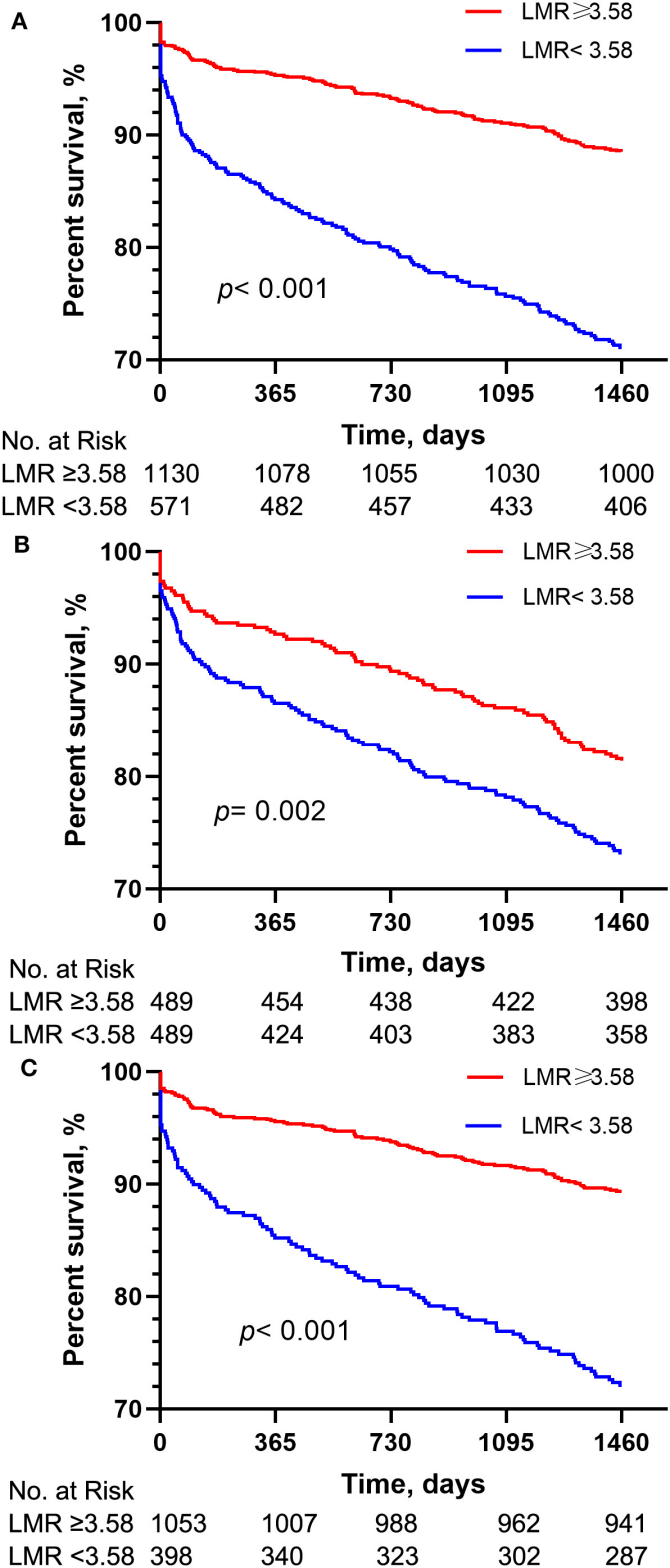
Kaplan-Meier survival analysis plot for 4-year overall survival. A significantly lower 4-year survival rate can be observed in the lower LMR group in patients before PSM **(A)**, patients with normal lymphocyte and monocyte counts **(B)** and patients after PSM **(C)**. *p-*value was calculated by log-rank test and indicated in the plot. LMR, lymphocyte-to-monocyte ratio; PSM, propensity score matching.

**Figure 2:**
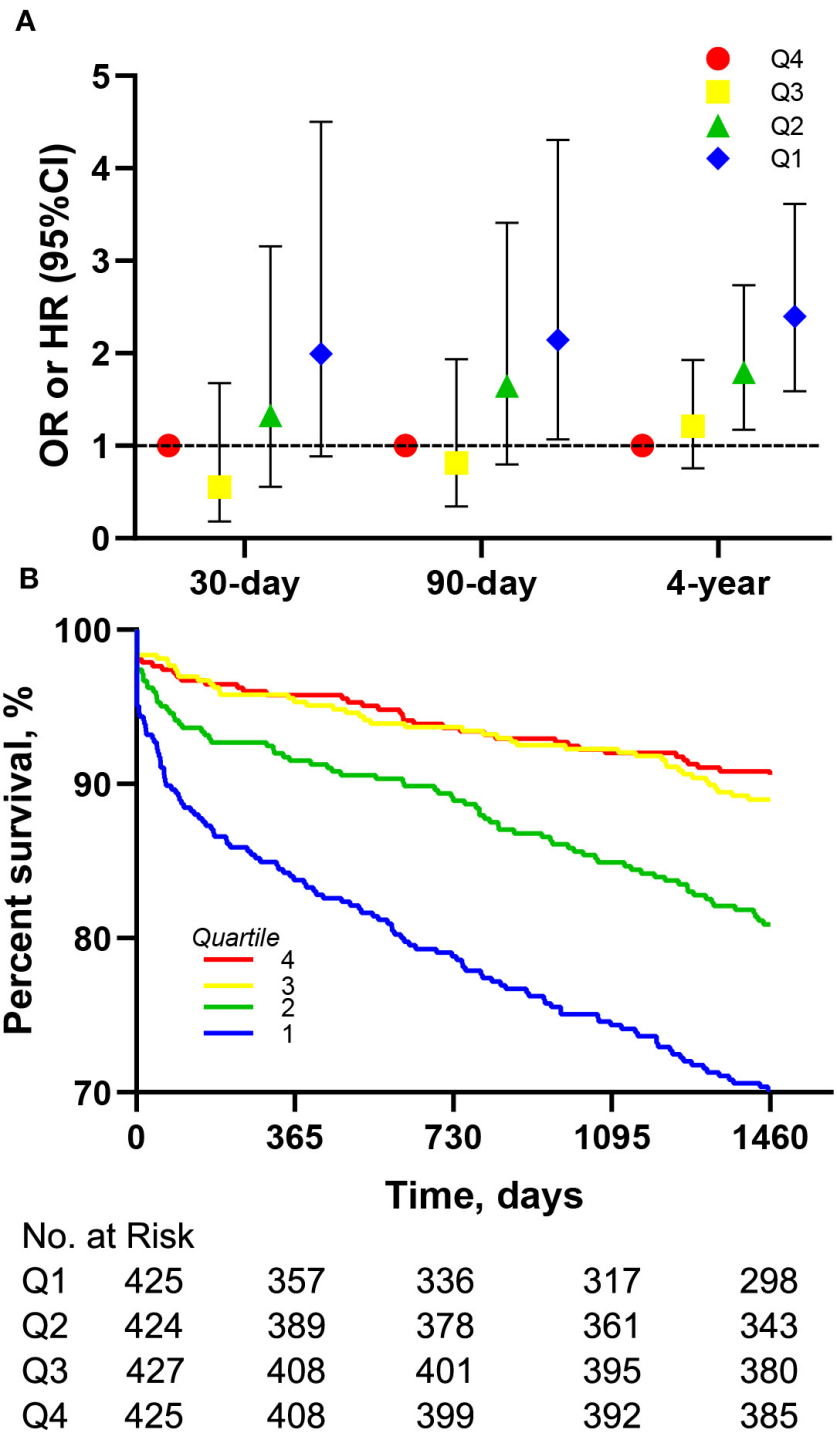
Association between different LMR quartiles and mortality in the entire cohort. **(A)** Association of different LMR quartiles with OR for 30-, 90-days mortality and HR for 4-year mortality. Models were adjusted for age, gender, congestive heart failure, cardiac arrhythmias, valvular disease, hypertension, renal failure and liver disease. **(B)** Kaplan-Meier survival analysis plot for 4-year overall survival in patients within different quartiles. LMR, lymphocyte-to-monocyte ratio; OR, odds ratio; HR, hazard ratio.

The Kaplan-Meier survival curves comparing the two groups are shown in Figure 1A. Patients with an LMR < 3.58 had a significantly lower 4-year survival rate compared to patients with an LMR ≥ 3.58 (71.7 vs. 88.5%, *p* < 0.001). In addition, the visualized adjusted HR of different LMR quartiles indicates that LMR was linearly correlated with higher risk of 4-year mortality. However, lower odds ratio of 30- and 90-days mortality was observed in patients within the second LMR quartile, despite the third and fourth quartiles still had higher mortality rate (Figure 2A). The Kaplan-Meier survival curves comparing patients within different LMR quartiles also demonstrated that patients with lower LMR were significantly associated with lower 4-year survival (*p* < 0.001) (Figure 2B).

A subgroup analysis of age (<70 years old; ≥70 years old), gender and comorbidities was used for further comparison of the long-term prognosis between these groups, and the results are presented in Table 4. The 4-year mortality rate were higher in groups with an LMR < 3.58 compared with the subgroups with an LMR ≥ 3.58, except for the subgroup of patients with liver disease (HR = 0.967, 95%CI: 0.363–2.65, *p* = 0.948). The interactions between the LMR and all subgroup factors were analyzed and significant interactions were observed only for CHF (*p* = 0.043). Female patients had a significantly higher risk of 4-year mortality with an LMR < 3.58 (HR = 2.629, 95%CI: 1.778–3.887, *p* < 0.001).

**Table 4:** Subgroup analysis for the effect of LMR on 4-year mortality in cardiac surgery patients.

**Subgroups**	**No. of patients**	**Mortality (%)**	**Lymphocyte-to-monocyte ratio < 3.58**		***p* for interaction**
			**HR (95%CI)**	***p***	
Age (years)					0.327
<70	1,004	110 (11.0)	1.840 (1.225–2.764)	0.003	
≥70	697	185 (26.5)	1.980 (1.460–2.684)	<0.001	
Gender					0.205
Female	523	118 (22.6)	2.629 (1.778–3.887)	<0.001	
Male	1178	177 (15.0)	1.664 (1.220–2.270)	0.001	
Liver disease					0.360
Yes	54	25 (46.3)	0.967 (0.353–2.650)	0.948	
No	1,647	270 (16.4)	2.061 (1.599–2.655)	<0.001	
Renal failure					0.175
Yes	166	65 (39.2)	1.611 (0.922–2.814)	0.094	
No	1,535	230 (15.0)	2.044 (1.563–2.673)	<0.001	
Hypertension					0.782
Yes	1,173	185 (15.8)	1.838 (1.346–2.511)	<0.001	
No	528	110 (20.8)	2.131(1.433–3.169)	<0.001	
Diabetes					0.710
Yes	585	115 (19.7)	1.471 (1.003–2.159)	0.048	
No	1,116	180 (16.1)	2.386 (1.738–3.276)	<0.001	
Chronic pulmonary disease					0.544
Yes	281	70 (24.9)	1.386 (0.847–2.268)	0.194	
No	1,420	225 (15.8)	2.114 (1.596–2.801)	<0.001	
Congestive heart failure					0.043
Yes	564	150 (26.6)	1.538 (1.094–2.164)	0.013	
No	1,137	145 (12.8)	2.374 (1.693–3.331)	<0.001	
Cardiac arrhythmia					0.420
Yes	836	193 (23.1)	1.986 (1.470–2.685)	<0.001	
No	865	102 (11.8)	1.935 (1.272–2.944)	0.002	
Heart valves disease					0.236
Yes	394	111 (28.2)	1.346 (0.899–2.016)	0.150	
No	1,307	184 (14.1)	2.429 (1.788–3.300)	<0.001	
CABG					0.991
Yes	1,227	207 (16.9)	1.933 (1.445–2.585)	<0.001	
No	474	88 (18.6)	2.027 (1.280–3.210)	0.003	

*HR (95%CI) were derived from Cox proportional hazards regression models and covariates were adjusted as in model 1 (Table 2)*.

*HR, hazard ratio; CI, confidence interval; CABG, coronary artery bypass grafting*.

### The Prognostic Significance of LMR in Patients With Normal Lymphocyte and Monocyte Counts

Considering a lower LMR may result from a reduced lymphocyte count or elevated monocyte count which may affect late mortality independently as reported previously, we also analyzed the association between the LMR and 4-year mortality in patients with normal lymphocyte and monocyte counts (*n* = 1,451). In the group with normal lymphocyte and monocyte counts, patients with an LMR < 3.58 were still at higher risk of prolonged ICU stay (3.0 vs. 2.2 days, *p* < 0.001), hospital mortality (4.77 vs. 1.52%, *p* < 0.001), 30-day mortality (6.53 vs. 1.71%, *p* < 0.001), 90-day mortality (9.3 vs. 2.8%, *p* < 0.001) and 4-year mortality (27.9 vs. 10.6%, *p* < 0.001) (Table 2).

As shown in Table 3, the results of multivariate Cox regression analysis in patients with normal lymphocyte and monocyte counts were similar as before. Patients with an LMR < 3.58 had significantly higher 4-year mortality compared to patients with an LMR ≥ 3.58 (Model 1: HR = 2.656, 95%CI: 2.032–3.471, *p* < 0.001; Model 2: HR = 2.052, 95%CI: 1.553–2.712, *p* < 0.001). As shown in Figure 1B, The survival curves comparing the two groups showed that, in the group with normal lymphocyte and monocyte counts, patients with an LMR < 3.58 also had a significantly lower 4-year survival rate compared to patients with an LMR ≥ 3.58 (72.1 vs. 89.4%, *P* < 0.001).

### The Prognostic Significance of LMR After PSM

Considering the imbalanced baseline characteristics between patients with an LMR < 3.58 and an LMR ≥ 3.58, we performed a 1:1 ratio PSM to balance the potential confounding factors, and 489 pairs of score-matched patients were generated. The baseline characteristics of patients after PSM are shown in Table 5. The demographics, vital signs, comorbidities and most laboratory events were well-balanced between these two groups. Since the lymphocyte, neutrophil and monocyte counts directly influenced the value of the LMR, we did not include them in the matched variables. After PSM, significant differences between the two groups can still be observed in ICU length of stay (3.0 vs. 2.9 days, *p* = 0.003), 90-day mortality (8.6 vs. 4.9%, *p* = 0.027) and 4-year mortality (26.8 vs. 18.6%, *p* = 0.002), but not significant in hospital mortality (3.48 vs. 2.66%, *p* = 0.458) and 30-day mortality (4.91 vs. 3.07%, *p* = 0.141) (Table 2).

**Table 5:** Baseline characteristics after propensity score matching (*n* = 978).

**Characteristics**	**LMR**		***p*-value**
	**<3.58 (*n* = 489)**	**≥3.58 (*n* = 489)**	
**Demographics**			
Age, years	68.7 (61–76.9)	69.7 (60.9–77.6)	0.482
Male, *n* (%)	356 (72.8)	373 (76.3)	0.218
Ethnicity, *n* (%)			0.051
White	372 (76.1)	353 (72.2)	
Black	8 (1.6)	17 (3.5)	
Others	109 (22.3)	119 (24.3)	
**Vital Signs**			
HR, beats/minute	85 (79.4–91.5)	85.1 (79.1–91.7)	0.992
SBP, mmHg	111.7 (106.3–120)	112.1 (106.2–120.1)	0.493
DBP, mmHg	56.2 (51.9–60.3)	55.9 (52.4–60.6)	0.699
RR, times/minute	16.9 (15.4–19.3)	16.8 (15–19)	0.202
Temperature, °C	36.9 (36.6–37.2)	36.9 (36.6–37.3)	0.561
SpO2, %	98.3 (97.3–99)	98.2 (97.4–99)	0.260
**Comorbidities**			
Congestive heart failure	201 (41.1)	202 (41.3)	0.999
Cardiac arrhythmias	283 (57.9)	286 (58.5)	0.886
Valvular disease	130 (26.6)	122 (24.9)	0.059
Hypertension	317 (64.8)	335 (68.5)	0.247
Chronic pulmonary disease	99 (20.2)	95 (19.4)	0.811
Renal failure	59 (12.1)	59 (12.1)	0.999
Liver disease	17 (3.5)	16 (3.3)	0.999
Coagulopathy	33 (6.7)	43 (8.8)	0.289
Diabetes	158 (32.3)	184 (37.6)	0.092
**Laboratory Events**			
LMR	2.7 (2.1–3.1)	4.5 (4–5.1)	<0.001
NLR	5.3 (3.6–8.5)	3.2 (2.4–4.3)	<0.001
PLR	182.2 (138–256.8)	128.1 (93.3–169)	<0.001
WBC, 10^9^/L	8.6 (6.5–11.4)	8.3 (6.7–10.6)	0.125
Neutrophils, %	76.3 (69.2–83.4)	70.1 (63.9–76.2)	<0.001
Monocytes, %	5.7 (4.4–7.1)	4.8 (3.8–5.8)	<0.001
Lymphocytes, %	14.5 (9.8–19.2)	21.9 (17.7–26.3)	<0.001
Platelet, 10^9^/L	221 (178–280)	222 (189–272.5)	0.757
Hemoglobin, g/dL	10.2 (9.3–11.2)	10.4 (9.4–11.2)	0.614
Serum sodium, mmol/L	137.5 (135.5–139)	137.5 (135.5–138.5)	0.613
Serum potassium, mmol/L	4.5 (4.2–4.9)	4.5 (4.3–4.9)	0.871
Serum creatinine, mg/dL	1 (0.8–1.3)	1 (0.8–1.2)	0.710
**Scores**			
SAPS II	35 (29–42)	34 (29–43)	0.857
SOFA	5 (3–7)	5 (3–7)	0.666
**Surgical type**			
CABG	331 (67.7)	374 (76.5)	0.002

*Values are presented as the mean ± standard deviation, median (interquartile range), or number of patients (%)*.

*HR, heart rate; SBP, systolic blood pressure; DBP, diastolic blood pressure; RR, respiratory rate; SPO2, percutaneous oxygen saturation; LMR, lymphocyte-to-monocyte ratio; NLR, neutrophil-to-lymphocyte ratio; PLR, platelet-to-lymphocyte ratio; WBC, white blood cell; SAPS II, Simplified Acute Physiology Score II; SOFA, Sequential Organ Failure Assessment; CABG, coronary artery bypass grafting*.

The results of multivariate Cox regression analysis in patients after PSM indicated that a LMR < 3.58 still remained an independent predictor of higher 4-year mortality (Model 1: HR = 1.568, 95%CI: 1.20–2.05, *p* = 0.001; Model 2: HR = 1.517, 95%CI: 1.159–1.986, *p* = 0.002) (Table 3). Additionally, the survival curves (Figure 1C) comparing the two groups showed that, after PSM, patients with an LMR < 3.58 still had a significantly lower 4-year survival rate compared to patients with an LMR ≥ 3.58 (73.2 vs. 81.4%, *P* = 0.002).

### Prognostic Predictive Ability of LMR

**Figure 3:**
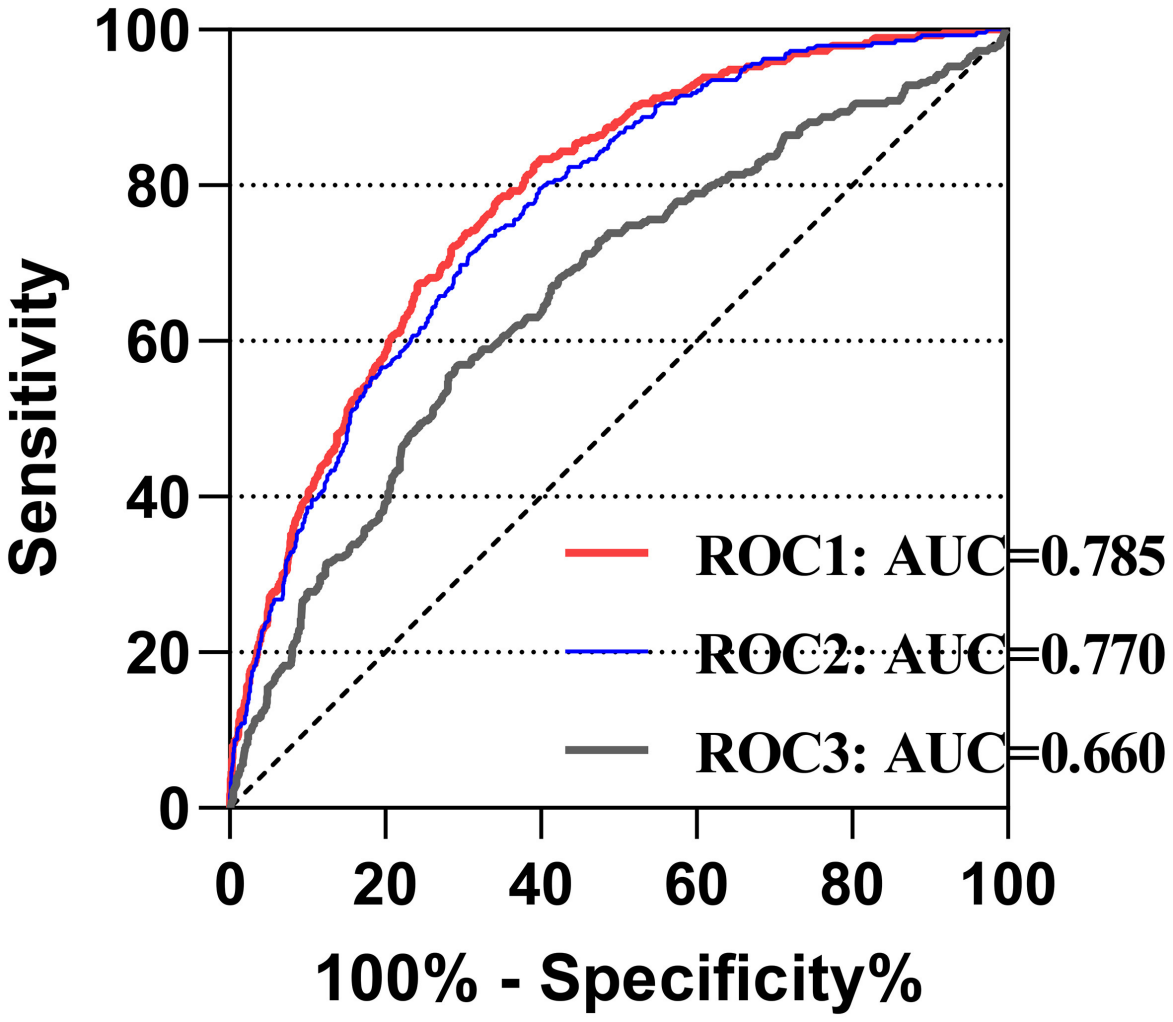
The receiver operating characteristic curves of predictive value of LMR for 4-year mortality in cardiac surgery patients. ROC1 included the LMR, age, gender, congestive heart failure, cardiac arrhythmias, valvular disease, hypertension, renal failure and liver disease; ROC2 included age, gender, congestive heart failure, cardiac arrhythmias, valvular disease, hypertension, renal failure and liver disease; ROC3 included only the LMR. LMR, lymphocyte-to-monocyte ratio; AUC, area under curve; ROC, receiving operating characteristic.

To assess the potential predictive role of the LMR for 4-year mortality and to evaluate the predictive model combining the LMR and other clinical variables, ROC curve analysis was performed, and the area under the curve (AUC) for LMR only was 0.660 (95% CI: 0.624–0.695; *p* < 0.001). For eight clinical variables in the model 1, the AUC was 0.770 (95% CI: 0.742–0.798; *p* < 0.001). After adding LMR, the AUC became 0.785 (95% CI: 0.758–0.812; *p* < 0.001), though without significant difference between two groups (*p* = 0.22) (Figure 3).

## Discussion

Our study investigated the association between LMR and risk of death among on-pump cardiac surgery patients within a cohort study with a 4-year follow-up. A PSM analysis was performed to balance the underlying confounding factors, and the results showed that lower preoperative LMR was a reliable predictor of unfavorable 4-year mortality. To our knowledge, this is the first study to demonstrate the correlation between the preoperative LMR and long-term mortality of cardiac surgery patients.

Inflammatory cells, including WBCs and their subtypes (lymphocytes, monocytes and neutrophils), have been well validated to play an indispensable role in cardiovascular diseases. Monocytes, as representatives of the innate immune system, play a vital role in the progression of atherosclerosis, and elevated monocytes are strongly associated with the development of coronary artery disease (2, 16, 17). Low lymphocyte counts have also been demonstrated to be an independent prognostic marker of heart failure and coronary artery disease (18, 19).

The potential mechanisms of the prognostic value of LMR are not fully understood now. We hypothesized that the ratio of certain WBC subtypes like LMR and NLR reflected the inflammatory background of patients, as assumed by Gibson et al. (20). The lymphocyte counts represent the general health state of patients rather than the direct protective role of lymphocytes for a certain pathophysiological process (3, 21). Monocytes, as the precursor of macrophages, reflect the balance between inflammation and immunity (22). Preoperative monocytosis represents the relatively insufficiency of activated macrophages therefore inflammation overweight immunity and leads to bad prognosis of cardiac surgery patients. The LMR can better integrate the clinical significance of lymphocytes and monocytes and provide additional prognostic value. Further studies are warranted to investigate the mechanism of the association between LMR and the prognosis in the future.

The LMR and NLR, which are novel markers defined by the ratio of WBC subtypes, have been shown in previous studies to have prognostic value with regard to cardiovascular outcomes (2, 3, 23). Silberman et al., after a 16-year follow-up, demonstrated that the NLR can predict both early and late outcomes in patients who underwent heart surgery (4). Lin et al. discovered the correlation between the LMR and in-hospital mortality of patients with acute type A aortic dissection (12). Oksuz et al. suggested that LMR levels were independent predictors of saphenous vein graft patency in patients who underwent CABG (13). Other studies also indicated that the LMR was associated with the prognosis of patients with heart failure, myocardial infarction and stable coronary artery disease (11, 24, 25).

The lower value of LMR may result from an elevated monocyte count or lower lymphocyte count. Both elevated monocytes and low lymphocytes are associated with a bad prognosis with regard to cardiovascular events, as reported before (2, 3, 23). Therefore, in order to discover whether the LMR itself can provide additional information, regardless of the elevation of monocytes and reduction of lymphocytes, we set a cohort which only included patients with normal lymphocyte and monocyte counts and discovered that a lower LMR was still associated with higher mortality in this cohort.

Another characteristic of our study was that we used the PSM analysis, which can minimize confounding factors in the baseline characteristics. The major results before and after PSM were basically consistent, but there were also differences that deserved further discussion. The HRs of an LMR <3.58 on the 4-year mortality before and after PSM differed from each other (Model 1: 2.656 vs. 1.568; Model 2: 2.052 vs. 1.517), which may be due to not only the balance of baseline characteristics but also the variation of the best cutoff value after PSM. Actually, due to the relatively small sample size and single-center-based cohort of the current study, the cutoff value of the LMR in our study may vary according to different strategies of matching. Further studies based on larger populations with external validation are warranted.

In our study, lower preoperative LMR was proven to be associated with a higher risk of long-term mortality. However, the results of ROC curve analysis showed that the AUC was 0.660 for the LMR only, which meant that a model containing only the LMR was not enough for prediction. Although LMR increased the AUC from 0.770 to 0.785 without significant difference, it can easily be acquired from routine blood examination, making it a simple and cost-effective indicator for prognostic evaluation. Furthermore, LMR might serve as an additional marker with the ability to provide information on preoperative inflammation and potentially increase the predictive accuracy of traditional models and risk scores, such as the EuroSCORE II (26). Unfortunately, since the MIMIC-III database lacked intraoperative information, we could not build a model containing all variables in the EuroSCORE II for comparison.

As shown in the results of the subgroup analysis, the LMR maintained its predictive capacity regardless of age, gender and most of the comorbidities. We found that compared with patients with CHF, a lower LMR value for patients without CHF may be more associated with a bad prognosis. CHF was one of the major adverse cardiovascular events (MACE), which was regarded as an important risk factor for long-term mortality for cardiac surgery patients. However, for patients without MACE, the predictive capacity of traditional models tended to be not as significant as patients with MACE. Our results demonstrated that a lower LMR had better predictive value in patients without CHF; therefore, it is reasonable to include the LMR in the models when considering the prognosis of patients without heart failure. Additionally, although a significant difference wasn't observed in the p for interaction, we noticed an interesting result that females had an ~2.6-fold higher risk of 4-year mortality with an LMR < 3.58, while males only a 1.6-fold higher risk of 4-year mortality. Further researches focusing on the correlation between sex and LMR are warranted in the future.

However, we had to admit that our study still had several limitations. First, the information on the WBC subtypes for some patients was missing or inappropriate for further analysis. Therefore, we directly deleted them in our cohorts, which might result in selection bias and this is one of the reasons why we chose the PSM analysis for balance. Second, considering the difference between test equipment and standards among various institutions, as well as our relatively small sample size and single-center-based cohort, we cannot deny the possibility that the best cutoff value may vary based on different study populations. Further studies and external validation based on multicenter large cohorts are required to determine the most appropriate cutoff value of the LMR according to different populations. Third, restricted by the contents of the MIMIC-III database, some important information, including inflammatory biomarkers, dynamic changes of the LMR, types of surgery, intraoperative data and postoperative complications, were recorded incompletely and not suitable for analysis.

## Conclusions

In conclusion, our findings showed, for the first time, that a lower LMR (<3.58) was correlated with a higher risk of death in on-pump cardiac surgery patients within a cohort study with a 4-year follow-up. The LMR is a potential prognostic predictor of the long-term mortality in cardiac surgery patients and may improve the predictive ability of traditional models or risk scores. Studies of large multicenter populations with longer follow-up are warranted for further validation.

## Data Availability Statement

The raw data supporting the conclusions of this article will be made available by the authors, without undue reservation.

## Ethics Statement

The studies involving human participants were reviewed and approved by the Massachusetts Institute of Technology (Cambridge, MA) and the Institutional Review Boards of Beth Israel Deaconess Medical Center (Boston, MA). Written informed consent for participation was not required for this study in accordance with the national legislation and the institutional requirements.

## Author Contributions

ML and JH: conception and design. ZW: administrative support. ZZ, SH, and RW: provision of study materials or patients. ZZ, ML, and HW: selection and assembly of data and data analysis and interpretation. All authors contributed to the article and approved the submitted version.

## Conflict of Interest

The authors declare that the research was conducted in the absence of any commercial or financial relationships that could be construed as a potential conflict of interest.

## References

[1] Wan S LeClerc JL Vincent JL. Inflammatory response to cardiopulmonary bypass: mechanisms involved and possible therapeutic strategies. Chest. (1997) 112:676–92. 10.1378/chest.112.3.676 9315800

[2] Ghattas A Griffiths HR Devitt A Lip GY Shantsila E. Monocytes in coronary artery disease and atherosclerosis: where are we now? J Am Coll Cardiol. (2013) 62:1541–51. 10.1016/j.jacc.2013.07.043 23973684

[3] Ommen SR Gibbons RJ Hodge DO Thomson SP. Usefulness of the lymphocyte concentration as a prognostic marker in coronary artery disease. Am J Cardiol. (1997) 79:812–4. 10.1016/S0002-9149(96)00878-8 9070569

[4] Silberman S Abu-Yunis U Tauber R Shavit L Grenader T Fink D. Neutrophil-lymphocyte ratio: prognostic impact in heart surgery. Early outcomes and late survival. Ann Thorac Surg. (2018) 105:581–6. 10.1016/j.athoracsur.2017.07.033 29132702

[5] Green J Bin Mahmood SU Mori M Yousef S Mangi AA Geirsson A. Stability across time of the neutrophil-lymphocyte and lymphocyte-neutrophil ratios and associations with outcomes in cardiac surgery patients. J Cardiothorac Surg. (2019) 14:164. 10.1186/s13019-019-0988-6 31511078 PMC6737616

[6] Engin M. Are pre and postoperative platelet to lymphocyte ratio and neutrophil to lymphocyte ratio associated with early postoperative AKI following CABG? Braz J Cardiovasc Surg. (2020) 35:239. 10.21470/1678-9741-2019-0482 32369308 PMC7199990

[7] Weedle RC Da Costa M Veerasingam D Soo AWS. The use of neutrophil lymphocyte ratio to predict complications post cardiac surgery. Ann Transl Med. (2019) 7:778. 10.21037/atm.2019.11.17 32042794 PMC6989975

[8] Liu Z Nguyen Khuong J Borg Caruana C Jackson SM Campbell R Ramson DM. The prognostic value of elevated perioperative neutrophil-lymphocyte ratio in predicting postoperative atrial fibrillation after cardiac surgery: a systematic review and meta-analysis. Heart Lung Circ. (2019) 29:1015–24. 10.1016/j.hlc.2019.11.021 32089488

[9] Bedel C Selvi F. Association of platelet to lymphocyte and neutrophil to lymphocyte ratios with in-hospital mortality in patients with type A acute aortic dissection. Braz J Cardiovasc Surg. (2020) 34:694–8. 10.21470/1678-9741-2018-0343 PMC6894039 31545575

[10] Kurtul A Duran M. The correlation between lymphocyte/monocyte ratio and coronary collateral circulation in stable coronary artery disease patients. Biomark Med. (2017) 11:43–52. 10.2217/bmm-2016-0179 27917651

[11] Silva N Bettencourt P Guimaraes JT. The lymphocyte-to-monocyte ratio: an added value for death prediction in heart failure. Nutr Metab Cardiovasc Dis. (2015) 25:1033–40. 10.1016/j.numecd.2015.07.004 26482565

[12] Lin Y Peng Y Chen Y Li S Huang X Zhang H. Association of lymphocyte to monocyte ratio and risk of in-hospital mortality in patients with acute type A aortic dissection. Biomark Med. (2019) 13:1263–72. 10.2217/bmm-2018-0423 31584289

[13] Oksuz F Elcik D Yarlioglues M Duran M Ozturk S Celik IE. The relationship between lymphocyte-to-monocyte ratio and saphenous vein graft patency in patients with coronary artery bypass graft. Biomark Med. (2017) 11:867–76. 10.2217/bmm-2017-0079 28976779

[14] Johnson AE Pollard TJ Shen L Lehman LW Feng M Ghassemi M. MIMIC-III, a freely accessible critical care database. Sci Data. (2016) 3:160035. 10.1038/sdata.2016.35 27219127 PMC4878278

[15] Camp RL Dolled-Filhart M Rimm DL. X-tile: a new bio-informatics tool for biomarker assessment and outcome-based cut-point optimization. Clin Cancer Res. (2004) 10:7252–9. 10.1158/1078-0432.CCR-04-0713 15534099

[16] van der Laan AM Hirsch A Robbers LF Nijveldt R Lommerse I Delewi R. A proinflammatory monocyte response is associated with myocardial injury and impaired functional outcome in patients with ST-segment elevation myocardial infarction: monocytes and myocardial infarction. Am Heart J. (2012) 163:57–65.e2. 10.1016/j.ahj.2011.09.002 22172437

[17] Maekawa Y Anzai T Yoshikawa T Asakura Y Takahashi T Ishikawa S. Prognostic significance of peripheral monocytosis after reperfused acute myocardial infarction:a possible role for left ventricular remodeling. J Am Coll Cardiol. (2002) 39:241–6. 10.1016/S0735-1097(01)01721-1 11788214

[18] Vaduganathan M Ambrosy AP Greene SJ Mentz RJ Subacius HP Maggioni AP. Predictive value of low relative lymphocyte count in patients hospitalized for heart failure with reduced ejection fraction: insights from the EVEREST trial. Circul Heart Fail. (2012) 5:750–8. 10.1161/CIRCHEARTFAILURE.112.970525 23051949

[19] Núñez J Miñana G Bodí V Núñez E Sanchis J Husser O. Low lymphocyte count and cardiovascular diseases. Curr Med Chem. (2011) 18:3226–33. 10.2174/092986711796391633 21671854

[20] Gibson PH Croal BL Cuthbertson BH Small GR Ifezulike AI Gibson G. Preoperative neutrophil-lymphocyte ratio and outcome from coronary artery bypass grafting. Am Heart J. (2007) 154:995–1002. 10.1016/j.ahj.2007.06.043 17967611

[21] Coller BS. Leukocytosis and ischemic vascular disease morbidity and mortality: is it time to intervene? Arterioscler Thrombos Vasc Biol. (2005) 25:658–70. 10.1161/01.ATV.0000156877.94472.a5 15662026

[22] Guilliams M Mildner A Yona S. Developmental and functional heterogeneity of monocytes. Immunity. (2018) 49:595–613. 10.1016/j.immuni.2018.10.005 30332628

[23] Guasti L Dentali F Castiglioni L Maroni L Marino F Squizzato A. Neutrophils and clinical outcomes in patients with acute coronary syndromes and/or cardiac revascularisation. A systematic review on more than 34,000 subjects. Thromb Haemostasis. (2011) 106:591–9. 10.1160/TH11-02-0096 21866299

[24] Kose N Akin F Yildirim T Ergun G Altun I. The association between the lymphocyte-to-monocyte ratio and coronary artery disease severity in patients with stable coronary artery disease. Eur Rev Med Pharmacol Sci. (2019) 23:2570–5. 10.26355/eurrev_201903_17406 30964185

[25] Fan Z Li Y Ji H Jian X. Prognostic utility of the combination of monocyte-to-lymphocyte ratio and neutrophil-to-lymphocyte ratio in patients with NSTEMI after primary percutaneous coronary intervention: a retrospective cohort study. BMJ Open. (2018) 8:e023459. 10.1136/bmjopen-2018-023459 30341133 PMC6196857

[26] Gummert JF Funkat A Osswald B Beckmann A Schiller W Krian A. EuroSCORE overestimates the risk of cardiac surgery: results from the national registry of the German Society of Thoracic and Cardiovascular Surgery. Clin Res Cardiol. (2009) 98:363–9. 10.1007/s00392-009-0010-8 19262978

